# Protocol for inducing deep vein thrombosis in C57BL/6J mice using the inferior vena cava stenosis model

**DOI:** 10.1016/j.xpro.2026.104665

**Published:** 2026-07-02

**Authors:** Aman Damara, Santiago Canon, Magdalena L. Bochenek, Katrin Schäfer, Fatemeh Shahneh

**Affiliations:** 1 Department of Dermatology, University Medical Center Mainz, Johannes Gutenberg University Mainz, 55131 Mainz, Germany; 2 Center for Thrombosis and Hemostasis (CTH), University Medical Center of the Johannes Gutenberg-University of Mainz, 55131 Mainz, Germany; 3 Department of Cardiology, Cardiology I, University Medical Center of the Johannes Gutenberg University Mainz, 55131 Mainz, Germany; 4 Research Center for Immunotherapy, University Medical Center, Johannes Gutenberg-University Mainz, 55131 Mainz, Germany

**Keywords:** Cell Biology, Immunology, Model Organisms, Molecular Biology

## Abstract

Here, we present a protocol for inducing deep vein thrombosis (DVT) in C57BL/6J mice using the inferior vena cava (IVC) stenosis model. We describe steps for surgical preparation, IVC ligation, and non-invasive detection and confirmation of thrombus development using high-frequency ultrasound (HFUS). We then detail procedures to dissect thrombi, prepare single-cell suspensions, and analyze infiltrating myeloid cells, including monocytes, by flow cytometry. This protocol allows precise timing, visualization, and immune profiling of early thrombus formation within 48 h.

For complete details and execution of this protocol, please refer to Shahneh et al.^1^^,^^2^

## Highlights

• Steps for surgical deep vein thrombosis induction in C57BL/6 mice using IVC stenosis
• Procedure for non-invasive thrombus detection using high-frequency ultrasound
• Guidance on dissecting thrombi and preparing single-cell suspensions
• Quantification of myeloid populations within thrombi using flow cytometry

## Abstract (editor highlights)

Publisher’s note: Undertaking any experimental protocol requires adherence to local institutional guidelines for laboratory safety and ethics.

## Graphical abstract

**Figure:**
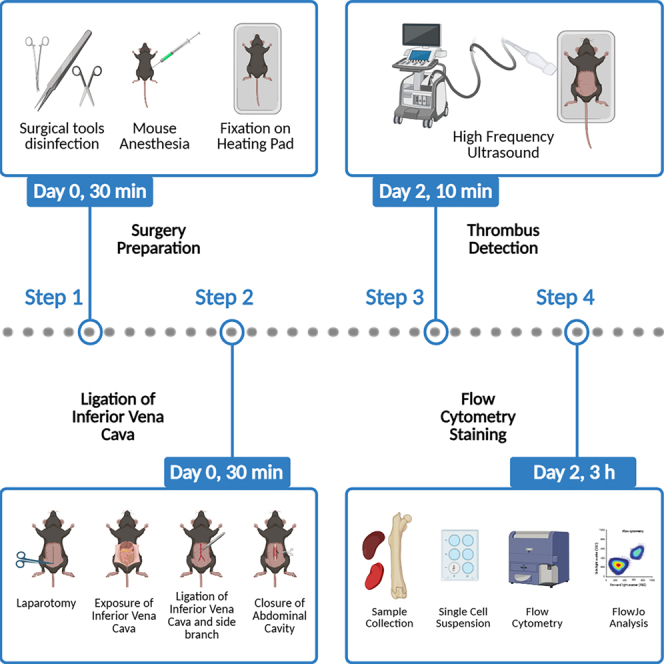

## Before you begin

Venous thrombosis is a major cause of cardiovascular morbidity, and mouse models have been essential for uncovering mechanisms driving clot formation and inflammation. This modified stenosis model enables reproducible thrombus formation within 48 h post-ligation, allowing controlled investigation of immune mechanisms in the formation^1^ and resolution^2^^,^^3^ of thrombus. Shahneh et al. showed that tissue factor (TF) competent Ly6C^hi^ monocytes enable thrombus formation in mice whereas specific inhibition of TF initiation complex on monocytes can prevent thrombus formation in preclinical model of systemic lupus erythematosus (SLE).^4^ Recently, Pekavayaz et al. found that the recruitment of neutrophils by non-classical monocytes induce thrombolysis in humans, establishing the concept of “immunothromobolysis”.^5^ The IVC stenosis model,^6^^,^^7^ along with right side branch ligation,^8^^,^^9^ induces thrombus formation through reduction of venous blood flow, thereby closely reproducing key clinical and histological features of human DVT, including rapid fibrin-rich thrombus formation, leukocyte recruitment, and early organization. Therefore, studying the myeloid population within the thrombus can aid in decoding the local inflammation patterns and devise therapeutic interventions.

In this protocol, we provide a detailed step-by-step approach to induce venous thrombosis, monitor clot development non-invasively using high-frequency ultrasound (HFUS), and isolate thrombi for downstream immunophenotyping. Flow cytometry is used to define and quantify myeloid cell populations within thrombi, with emphasis on monocyte and neutrophils characterization at day 2 post-stenosis. Together, this integrated workflow provides a robust platform for interrogating inflammatory cell contributions to DVT initiation and propagation.

### Innovation

This detailed protocol enables understanding of the myeloid cell recruitment at the time of thrombus formation via a streamlined workflow of thrombus induction, detection, isolation, and staining. This modified IVC stenosis model, with right side branch ligation, enables reproducible experimental DVT studies. We show 2D and 3D representation of thrombus clearly depicting the clot formation, ligation, and blood flow. This protocol can aid in temporal studies of thrombus formation and resolution, and thus contributing to studying immunological impact of coagulation proteases.

### Institutional permissions

All mouse experiments were performed in accordance with institutional and national regulations for animal welfare issued by the Federal State of Rhineland-Palatinate (Germany). Protocols were approved by the State Investigation Office (authorization numbers G22-1-022). All procedures were conducted under strict adherence to ethical guidelines, and every effort was made to minimize animal suffering.

### Surgical bench preparation

**Timing: 30 min**

**Figure 1:**
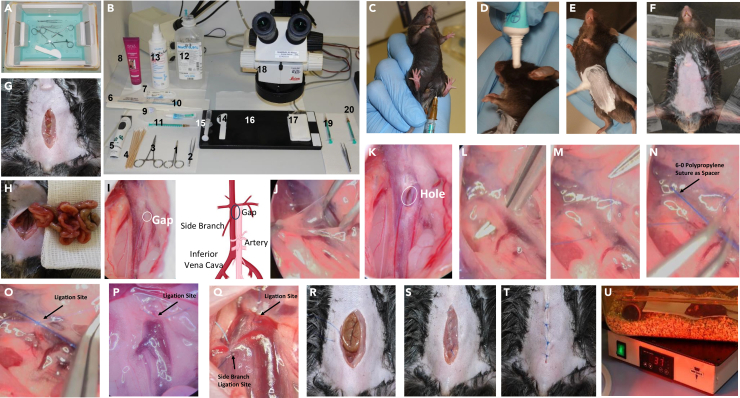
Step-by-step modified inferior vena cava (IVC) stenosis ligation (A) Surgical instruments in anti-septic solution. (B) Surgical bench setup. [1- Iris scissor. 2- Curved micro-dissecting forceps. 3- Locking forceps. 4- Cotton swabs. 5- Hair trimmer. 6- Polypropylene 8-0 suture. 7- Polypropylene 6-0 suture. 8- Hair removal cream. 9- Surgical tape. 10- Eye ointment. 11- Antidote syringe. 0.9% NaCl isotonic solution. 13- Antiseptic solution. 14- Hair removal spatula. 15- 5mL syringe (warm NaCl solution). 16- Heating pad. 17- Gauze. 18- Microscope. 19- Analgesia syringe. 20- Anesthesia syringe]. (C) Anesthesia injection. (D) Eye ointment application. (E) Hair removal cream application. (F) Fixation on heating pad. (G) Abdominal and peritoneum incision. (H) Externalization of intestines. (I) Gap between artery and IVC. (J) Making a hole between artery and IVC. (K) Hole between artery and IVC. (L) Pass micro-forceps under the IVC. (M) Pass 8-0 suture under the IVC. (N) Put spacer on IVC. (O) Two-single knot ligation of IVC. (P) Ligated IVC. (Q) Side branch ligation. (R) Internalization of intestines. (S) Muscle layer closure. (T) Epidermal layer closure. (U) Post-operative care under infrared lamp.

***Note:*** Sterilization in disinfectant solution should be at least 20 min.

1. Sterilize all surgical tools (Hemostat, curved micro 2000 forceps, Iris scissors) in disinfectant solution. It is also advised to autoclave before surgery (Figure 1A).

2. Wipe the work bench and heating pad with 70% ethanol or disinfectant solution.
3. Turn on the heating pad and adjust its temperature to 37°C.
4. Organize all necessary items such as surgical tools, gauze, hair trimmer and removal cream, NaCl isotonic solution, cotton swabs, eye ointment, spatula to remove hair cream, antiseptic spray, antidote and analgesia injections, surgical tape, and 6–0 and 8–0 polypropylene sutures as per convenience (Figure 1B).

***Note:*** Organizing the workspace according to your dominant hand streamlines the surgical process. Label syringes as anesthesia, antidote, and analgesia to avoid wrong injection during surgery.

5. Fill a 5 mL syringe with NaCl solution and keep it on the heating pad to warm it.

***Note:*** Warming NaCl helps prevent hypothermia in mice.

### High-frequency ultrasound setup

**Timing: 10 min**

6. Warm the ultrasound gel to 37°C using a water bag or water bath.
7. Set the heating pad to 37°C before anesthetizing mice.
8. Confirm adequate supply of isoflurane and oxygen.
9. Attach the MX550D transducer to the Vevo3100® machine and turn it on. Select the “Mouse (large abdominal)” scanning option, start a new study, and label appropriately.

## Key resources table

**Table:** 

REAGENT or RESOURCE	SOURCE	IDENTIFIER
**Antibodies**		

CD11b (1:200)	BioLegend	Cat# 101212; RRID:AB_312795
CD3 (1:400)	BioLegend	Cat# 100233; RRID:AB_2561387
CD45R (1:400)	BioLegend	Cat# 103248; RRID:AB_2650679
NK1.1 (1:400)	BioLegend	Cat# 108737; RRID:AB_2562216
CD45 (1:200)	BioLegend	Cat# 103147; RRID:AB_2564383
Ly6C (1:200)	Thermo Fisher Scientific	Cat# 25-5932-82, RRID:AB_2573503
Ly-6G (1:200)	BioLegend	Cat# 127606; RRID:AB_1236494
F4/80 (1:200)	BioLegend	Cat# 123132; RRID:AB_11203717
CD11c (1:200)	BioLegend	Cat# 117328; RRID:AB_2129641
CD16/32 (1:50)	Thermo Fisher Scientific	Cat# 14-0161-82; RRID:AB_467133
Fixable Viability Dye (1:800)	Thermo Fisher Scientific	Cat# 65-0866-14

**Biological samples**		

Mouse Spleen	C57BL/6 J	Strain#:000664; RRID:IMSR_JAX:000664
Mouse Bones	C57BL/6 J	Strain#:000664; RRID:IMSR_JAX:000664
Mouse Thrombus	C57BL/6 J	Strain#:000664; RRID:IMSR_JAX:000664

**Chemicals, peptides, and recombinant proteins**		

Fentanyl	Received from Translational Animal Research Center (TARC)	https://www.unimedizin-mainz.de/tarc/index.html
Midazolam	ratiopharm	Reg# 44856.01.00
Dorbene Vet	Zoetis	Reg# 401062.00.00
Flumazenil	Inresa Arzneimittel GmbH	Reg# 60952.00.00
Alzane	Zoetis	Reg# 401303.00.00
Bupresol Vet	Cp Pharma	Reg# 402614.00.00
10× RBC Lysis Buffer	BioLegend	Cat# 420302
1× Dulbecco PBS	Sigma-Aldrich	Cat# D8537-500ML
FCS	Merck	Cat# F4135-500ML
Disodium EDTA	Carl Roth	Cas# 6381-92-6
Sonosid® Ultrasound Gel	Asid Bonz GmbH	Ref# 782010
Bepanthen® Augen- und Nasensalbe	Bayer Vital GmbH	Reg# 6029009.00.00
Snä Epil Enthaarungs- creme	Rufin Cosmetic GmbH	Art# 28612

**Experimental models: Organisms/strains**		

C57BL/6 J (male and female, 8-12 weeks)	The Jackson Laboratory	Strain#:000664; RRID:IMSR_JAX:000664

**Software and algorithms**		

FlowJo	BD	https://flowjo.com/
Vevo Lab	Fujifilm Visualsonics	https://www.visualsonics.com/product/software/vevo-lab
FACSDiva™	BD	https://www.bdbiosciences.com/en-de/products/software/instrument-software/bd-facsdiva-software

**Other**		

Prolene™ Suture 6-0	Johnson&Johnson MedTech	Ref# 8889H
Prolene^TM^ Suture 8-0	Johnson&Johnson MedTech	Ref# EH7469LG
Micro 2000 Forceps	Medicon	Ref# 07.61.25
Fine Hemostat Straight	Fine Science Tools	Part# 13007-12
Extra Narrow Scissors	Fine Science Tools	Part# 14088-10
Corning® Falcon® Cell Strainer 70 μm	Merck	CLS352350
Corning® Falcon® Cell Strainer 40 μm	Merck	CLS352340

## Materials and equipment

**Table:** Anesthesia Cocktail

Drug	Stock Concentration (mg/mL)	Working Concentration (mg/mL)	Volume of stock solution (μL)
Fentanyl	0.5	0.003125	6.25
Midazolam	5	0.3125	62.5
Dorbene Vet	1	0.03125	31.25
NaCl 0.9%	N/A	N/A	900

Store at 4°C, protected from light, for up to one day.

**CRITICAL:** Follow the dosage as approved by the state investigation office.

**Table:** Antidote Cocktail

Drug	Stock Concentration (mg/mL)	Working Concentration (mg/mL)	Volume of stock solution (μL)
Flumazenil	0.1	0.0625	625
Atipamezole	5	0.3125	62.5
NaCl 0.9%	N/A	N/A	312.5

Store at 4°C, protected from light, for up to one day.

### Analgesia cocktail

Add 83 μL from Buprenorphine (Stock concentration 0.3 mg/mL) to 917 μL of 0.9% NaCl to reach the working concentration (0.025 mg/mL). Store at 4°C, protected from light, for up to one day.

**Table:** FACS Buffer

Reagent	Working Concentration	Volume (mL)
1× DPBS	N/A	970
FCS	2%	20
0.2 M EDTA	2mM	10

Store at 4°C for up to one week.

### RBC lysis buffer

Dilute 10× RBC Lysis buffer to 1× with room temperature deionized water. Store at 4°C, protected from light, for up to 1 month when sterile-filtered.

## Step-by-step method details

### Inferior vena cava ligation

**Timing: 1 h**

This step describes the step-by-step details for ligating the IVC and the lateral right side branch to induce thrombus formation in mice.

1. Animal preparation and anesthesia.
  a. Calculate the anesthesia injection volume based on the mouse body weight (Fentanyl 0.025 mg/kg, Midazolam 2.5 mg/kg, Dorbene Vet 0.25 mg/kg) and inject intraperitoneally (Figure 1C).
    **CRITICAL:** Anesthesia can impair pulmonary function, causing heavy breathing. Pinching or gently moving the mouse often restores normal breathing. Do not inject additional anesthesia as it can collapse the lungs.
    ***Note:*** If unsure about anesthesia sensitivity, inject 75% of the calculated dose first. Check anesthesia depth before injecting the remaining 25%. Do not exceed 125% of the total dose. If surgery lasts longer than 30 min, inject 50% of the calculated dose again.
    ***Optional:*** Analgesia before surgery is recommended; however, in our protocol the anesthesia cocktail contains fentanyl which provides analgesic effect.
    ***Optional:*** Antibiotics may be used by beginners to reduce microbial contamination risk. Properly performed surgeries should not require antibiotics.
    ***Optional:*** Alternatively, isoflurane can also be used to maintain anesthesia. Perform chamber anesthesia with isoflurane (3%–4%) and oxygen (0.8–1 L/min) before switching to nose cone. Isoflurane can be reduced to 1%–2% and oxygen to 0.5–1 L/min.
  b. Confirm anesthesia by checking pedal reflex (pinch skin between toes), ensuring regular breathing, and pink mucous membranes at nose and mouth indicating adequate oxygenation.
  c. Apply eye ointment as a thin layer (approximately a pinhead-sized amount, volume ≈ 1–5 μL) to prevent corneal damage (Figure 1D).
  d. Remove abdominal hair using a trimmer followed by application of hair removal cream (Figure 1E).
    i. Leave for a few minutes and remove cream/hair with a spatula.
    ii. Spray skin with antiseptic solution.
      ***Note:*** Shave at least three times more area than immediately required for surgery. Shaving the entire abdomen is recommended for improved thrombus visualization during HFUS later.
  e. Fix mice on the heating pad with surgical tape, abdomen facing upwards (Figure 1F).
    **CRITICAL:** The surgeon should spray gloves with 70% ethanol before beginning the surgery, ideally change gloves between surgeries, work within the sterile field, and avoid touching non-sterile surfaces.
2. Laparotomy and exposure of inferior vena cava.
  a. Lift the abdominal skin with forceps and make a vertical incision approximately 2 cm long using surgical scissors.
  b. Make a second vertical incision in the peritoneum along the linea alba (Figure 1G).
    **CRITICAL:** It is essential to lift the skin with forceps to avoid damaging internal organs during incision. In the peritoneum, the linea alba (white line) is thinner and more transparent. Incising to the left or right can make wound closure difficult, as this layer can rupture easily.
  c. On the mouse’s right-hand side, pass the suture through the skin folds and secure the suture with a hemostat to keep the skin retracted.
  d. Moisten the tip of cotton swabs with warm NaCl solution and gently externalize the intestines between gauze folds, keep them continuously moist by adding warm NaCl solution every five minutes until wound closure (Figure 1H).
    **CRITICAL:** Rough handling can rupture small vessels leading to blood loss.
  e. Use moistened cotton swabs to move peritoneal fat aside and clearly expose the inferior vena cava (Figure 1I).
    ***Note:*** Keeping cotton swabs moist reduces friction between mouse tissues and helps prevent mechanical trauma.
3. Ligation of inferior vena cava.
  **CRITICAL:** Perform all steps under the microscope at 2.5× magnification. Do not attempt IVC ligation without magnification.
  a. Identify the small gap between the inferior vena cava and the artery. This is usually located below the left renal vein and appears as a small fatty connective tissue region (Figure 1I).
    ***Note:*** Use moistened cotton swabs to identify this location. The IVC and artery are closely attached via fascia. Just below the left renal vein, the fascia is thicker, creating a small gap between the two vessels.
    ***Note:*** Artery wall is pale pink to white in color that blends in with the fat tissue. Look closely to identify the anatomy before proceeding to the next step.
    **CRITICAL:** Do not use forceps at this stage as the vein wall is extremely thin and punctures can cause major blood loss.
  b. Prepare the 8–0 polypropylene suture and keep it within immediate reach.
    ***Note:*** Pre-cut the suture length to a standardized size (e.g. 4–5 cm) before each surgery to reduce handling time inside the cavity.
  c. Take micro-dissecting forceps in each hand. Using your left hand, elevate the IVC by holding the surrounding fat tissue to the right side of the vessel (opposite to left renal vein). Using your right-hand forceps, make a hole at the defined location using gentle continuous opening and closing motions (Figures 1J and 1K, Methods Video S1)
    **CRITICAL:** Since the color of artery wall and fatty tissue blend in, failure to differentiate between them can lead to artery damage.
    ***Note:*** Do not damage the fat tissue lateral to the IVC, as it supports and stabilizes the vessel while forming the passage.
    Methods Video S1. Protocol video showing the IVC ligation, related to step 3
  d. While keeping the vein lifted, pass the right-hand forceps through the hole and from underneath the IVC, and emerge on the right side through the fat tissue (Figure 1L).
  e. Using the left-hand forceps, transfer the 8–0 polypropylene suture to the mouth of the right-hand forceps.
  f. Gently pull the right-hand forceps back through the hole and exit from below the IVC, bringing the suture through to both sides (Figure 1M).
  g. Place a space holder (6–0 polypropylene suture) on top of the vein wall surface. Make 2 single knot ligations over the space holder (Figures 1N and 1O).
    ***Note:*** Use the same spacer consistently to standardize degree of stenosis between operators.
  h. Remove the spacer gently (Figure 1P).
    ***Note:*** Confirm stenosis before closure. The IVC becomes visibly distended due to reduced venous flow. (troubleshooting 1).
4. Ligation of side branch.
  ***Note:*** Identify and document all visible lateral side branches in both the sexes. In female mice, treat the right ovarian vein draining into the IVC within the operative segment as a lateral side branch. In male, treat the right gonadal vein equivalently.
  **CRITICAL:** Branches within approximately 1–1.5 mm of the ligature can markedly reduce or prevent thrombus formation.^10^^,^^11^ Ligate them to ensure consistent flow restriction. Do not ligate posterior branches.
  a. Create a hole in the close vicinity of lateral right side branch and pass the suture from under as explained in the previous section.
  b. Make 2 single knot ligations and confirm the ligation (Figure 1Q).
    ***Note:*** There is a chance to include the back muscles and adipose tissue within the suture along with the side branch. This can affect the tightness of ligation and thereby reduced efficiency in hindering the blood flow.
5. Closure of abdominal cavity.
  a. Lift the sutured skin edge and gently return the moist intestines back into the abdominal cavity using a cotton swab (Figure 1R).
  b. Add 0.5–1 mL of warm NaCl solution into the abdominal cavity to prevent dehydration.
    ***Note:*** The required volume depends on mouse size. Too much volume can make closure of the abdominal muscle layer and skin difficult.
  c. Ligate the abdominal muscle layer with 6–0 polypropylene filament using a continuous suture technique (Figure 1S).
    ***Note:*** Since the abdominal muscle layer is covered by epidermis, continuous suturing can be performed efficiently.
  d. Close the external epidermal layer using simple interrupted sutures. Make 3 double knots to ensure complete closure. Adjust the number of sutures according to the length of incision (Figure 1T).
    **CRITICAL:** Strong and complete skin closure is essential to prevent wound opening. Mice, especially males, can scratch the abdomen and open weak ligations.
  e. Spray the ligated wound area with antiseptic solution.
  f. Inject the analgesia subcutaneously at 0.1 mg/kg body weight.
  g. Inject antidote cocktail intraperitoneally with Flumazenil at 0.5 mg/kg body weight and atipamezole at 2.5 mg/kg body weight working concentration.
    ***Note:*** Record exact time of antidote/analgesia administration to standardize postoperative analgesia interval for all animals.
6. Post-operative care.
  a. Prepare a recovery cage with a flat paper bedding and place it on a heating plate set to 37°C.
  b. Transfer the mice into the recovery cage, ideally on a flat paper bedding, using clean paper towels (Figure 1U).
    ***Optional:*** Position an infrared lamp at approximately 2 feet.
    **CRITICAL:** Do not position the infrared lamp too close, as this can overheat the cage and lead to dehydration. Mice typically do not drink water while recovering from anesthesia.
  c. Transfer the mice back to the original cage once it appears stable and awake.
    **CRITICAL:** Identify critical pain indicators prior to return.
  d. Continue injecting analgesia every 4 h during the light phase (a total of no more than 3 times per day, but at least 2 times on the day of surgery). (troubleshooting 2)
  e. To ensure adequate analgesia during the dark phase, administer buprenorphine overnight via drinking water (concentration: 0.009 mg/mL).
    ***Note:*** Monitor mice for at least one week post-surgery until normal behavior (interaction, feeding, drinking) resumes, using a standardized score sheet (Table S1).
    **CRITICAL:** On Day 0, monitor during recovery from anesthesia and 4–6 h post-surgery alongside the second buprenorphine dose. On Days 1 and 2, monitor twice daily. Thereafter, monitor at least once daily if clinical signs persist until absent for ≥24 h.
    ***Note:*** Assess pain based on established indicators, including orbital tightening, nose or cheek bulge, drooped ears, raised whiskers, hunched posture, reduced appetite, and social avoidance.

### High-frequency ultrasound

**Timing: 10 min**

This step describes the non-invasive detection of thrombus using high frequency ultrasound at day 2 after IVC stenosis.

7. Animal Preparation.
  a. Place the mice in the anesthesia box with 2.5% isoflurane and 100% oxygen and keep the cage on a 37°C heating plate.
    **CRITICAL:** Avoid leaving the mice in the anesthesia box for extended periods to prevent hypothermia.
  b. Transfer and fix the mouse onto the heating pad with the nose secured in the anesthesia nose cone. Monitor temperature and heart rate (Figure 2A).
    ***Note:*** Isoflurane concentration can be reduced to 1%–1.5% after stable anesthesia.

**Figure 2:**
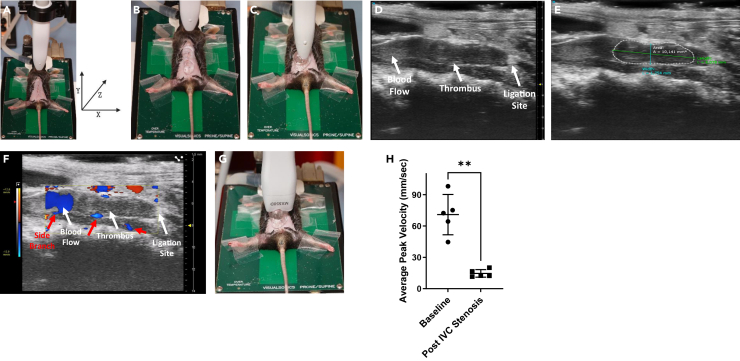
Step-by-step high frequency ultrasound protocol (A) Mice fixation in anesthesia cone. (B) Apply ultrasound gel. (C) Ultrasound acquisition. (D) 2D thrombus image with ligation site and thrombus shown. (E) Measurement of thrombus length, width, and area. (F) Thrombus image in color doppler mode. (G) Scan head position for 3D acquisition. (H) Assessment of average blood flow velocity in the IVC in response to partial ligation (n = 5). Statistical significance was determined by unpaired *t* test. Data are presented as mean ± SD. P value < 0.05 was considered statistically significant.
  c. Ensure the abdomen is completely hair-free. Shave again if needed:
    ***Note:*** Hair produce signal artifacts in ultrasound imaging.
8. Data Acquisition.
  a. Apply warm ultrasound gel to the abdomen and scan with the transducer by moving in X, Y or Z planes (Figure 2B).
    ***Note:*** Transmitting frequency should be 40 MHz.
  b. To confirm the location of the thrombus, use pulse-wave Doppler mode to identify abdominal aorta flow by moving transducer along the X-axis and then track the adjacent ligated IVC (Figure 2C)
    ***Note:*** Z-axis is parallel to mouse. X-axis is horizontally perpendicular. Y-axis is vertically perpendicular.
  c. The vein narrows at the ligation site and a cylindrical thrombus is visible towards the posterior side (Figures 2D, Methods Video S2).
    Methods Video S2. 2D video of thrombus in B-mode, related to step 8
  d. Press gently in the Y-plane to confirm solidity; a solid thrombus does not compress.
  e. Using Vevo lab software, you can visualize and calculate area, length and width of the thrombus (Figure 2E).
  f. Using color Doppler ultrasound mode, you can visualize blood flow within the vein (appears as blue) but also in side branches (marked with red arrows) (Figures 2F, Methods Video S3).
    Methods Video S3. 2D video of thrombus in color doppler mode, related to step 8
  g. To record venous thrombus in 3D environment, move the scan-head by 90 degrees to the left, that it is now in X-view (Figure 2G). Choose correct motor position for a “start” and “stop”.
    ***Note:*** Try to visually control if you are still on the top of the thrombus. For more stable images taken between the heartbeats, you can use respiratory gating.
  h. Scan the required number of frames and export as 3D recording.
    ***Note:*** If the image is unclear, apply additional warm gel or adjust the transducer angle. Tilting the posterior side of the heating pad often improves imaging quality.
  i. Using pulsed-wave (PW) Doppler ultrasound mode, you can quantify blood flow velocity at the IVC ligation site by measuring peak velocities before (Methods Video S4) and after (Methods Video S5) partial ligation.
    ***Optional:*** Take an average of at least 5 cardiac peak velocities before and after partial ligation to assess degree of stenosis (Figure 2H).
    Methods Video S4. Representative pulsed-wave doppler recording acquired at the IVC prior to partial ligation, showing high velocity, pulsatile blood flow, related to step 8
    Methods Video S5. Representative pulsed-wave doppler recording acquired at the ligation site following partial ligation, demonstrating low velocity, blunt doppler waveforms indicative of reduced forward flow, related to step 8
  j. Remove the mouse from the heating pad, wipe off gel, and place under infrared light for 5–10 min prior to recovery.
  k. Use Vevo Software to export either still images or 2D videos demonstrating thrombus residing Vena cava but also prepare 3D reconstruction of the thrombus either with the data as a surface (Methods Video S6) or just thrombus (Methods Video S7), as published earlier.^12^^,^^13^
    Methods Video S6. 3D reconstruction of abdomen showing thrombus with surface data included, related to step 8
    Methods Video S7. 3D reconstruction of thrombus, related to step 8

### Tissue harvesting

**Timing: 30 min**

Here, we describe the collection of spleen, thrombus, and bone samples.

9. Animal Preparation.
  a. Euthanize the mouse via CO_2_ asphyxiation followed by cervical dislocation.
  b. Confirm death and pin the mouse in a supine position.
  c. Sterilize the abdomen with 70% ethanol.
10. Spleen Harvesting.
  a. Make a midline incision through the skin and peritoneum using sterile scissors.
  b. Locate the spleen (dark red organ located on the left side of the abdominal cavity near the stomach).
  c. Carefully excise the spleen using forceps and scissors, avoiding excessive force.
  d. Remove surrounding fat and connective tissue.
  e. Wash the spleen once in cold 1× PBS.
  f. Place spleen into a tube containing FACS buffer. Keep it on ice.

***Note:*** Minimizing time between tissue removal and processing can yield better single cell suspension and downstream assays.

11. Thrombus Harvesting.
  **CRITICAL:** Perform thrombus harvesting under the microscope.
  a. Extend the midline incision caudally toward the pelvic region to fully expose the abdominal cavity.
  b. Remove intestines gently.
  c. Locate the ligation site and identify the thrombus toward the caudal end (Figure 3A).
    i. Remove all the fibrotic tissue around the IVC.
    ii. Clear the vein well below the thrombus and fibrotic region.
    iii. Remove lymph nodes to avoid cell contamination.
      **CRITICAL:** Lymph nodes are small and can adhere to the vein wall/thrombus. Microscope dissection is essential.

**Figure 3:**
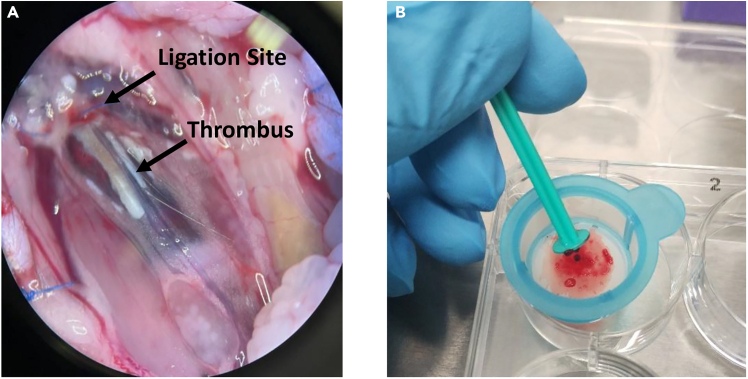
Thrombus formation and processing for flow cytometric analysis (A) Thrombus formation at Day 2. (B) Thrombus processing on cell strainer.
  d. Hold the vein gently with curved micro-dissecting forceps from the lower end distal to the ligation site and lift it upward.
  e. Cut the tissue below the vein and advance cuts toward the ligation site.
  f. Carefully cut above the ligation site and remove the cut vein segment.
  g. Hold the vein at the ligation site and gently express the thrombus from the distal end.
    **CRITICAL:** Prevent mechanical disruption of the thrombus if downstream imaging/weighing/length measurement is required.
  h. Transfer the thrombus to the tube containing FACS buffer and store on ice until processing.
12. Bone Harvesting.
  a. Harvest intact femur and tibia by making cuts parallel to the leg anatomy.
    ***Note:*** Harvest femur by making an incision close to the backbone. Do not cut the bone from the middle.
  b. Remove the skin and muscles before transferring the bones to ice cold FACS buffer tube.
    ***Note:*** Collect bones from both the legs and the hands to ensure sufficient viable cell numbers.

### Tissue processing

**Timing: 30 min**

Here, we describe the processing of spleen, thrombus, and bone samples into single cell suspension.

13. Spleen and Thrombus Processing.
  ***Note:*** Both spleen and thrombus processing follow the same steps.
  a. Remove spleen/thrombus sample from ice and place it onto a 70 μm cell strainer positioned over a 6-well plate well.
  b. Gently dissociate the spleen/thrombus through the strainer using the back end of a syringe plunger (Figure 3B).
  c. Rinse the strainer with 3–4 mL FACS buffer.
  d. Transfer the cell suspension into a 15 mL Falcon tube and top up volume to 15 mL with FACS buffer.
  e. Centrifuge at 350 ×*g* for 10 min at 4°C.
  f. Decant supernatant into biohazard waste and proceed to RBC lysis.
    i. Add 1 mL RBC lysis buffer and incubate on ice for 10 min.
    ii. Stop lysis reaction by filling the tube with FACS buffer.
    iii. Centrifuge at 350 ×*g* for 10 min at 4°C.
  g. Resuspend the pellet in 2 mL FACS buffer and strain through a 40 μm filter to remove extracellular debris and fat aggregates.
  h. Centrifuge again at 350 ×*g* for 10 min at 4°C.
  i. Resuspend the final cell pellet in FACS buffer, keep on ice, and count cells before proceeding to flow cytometry staining.
14. Bone Processing.
  a. Take 0.5 mL centrifuge tube and make a hole at the bottom with a 19 gauge needle.
  b. Place this tube in an empty 1.5 mL Eppendorf tube.
  c. Take the bones from ice-cold FACS buffer. Make sure it is completely clean from mouse tissues.
  d. Cut the bone from the end that appear whiter.
    ***Note:*** The whiter side of bone contains fewer cells and the cut acts as a passage for the cells to pass through.
  e. Place the cut bone in the 0.5 mL Eppendorf tube. The cut end facing downwards.
  f. Centrifuge the 1.5 mL tube, bearing the 0.5 mL tube with cut bones, at 10,000 *g* for 10 seconds at 4°C.
  g. The bone marrow collects in the 1.5 mL tube. Transfer the cells to 15 mL Falcon tube and wash with 10 mL FACS buffer.
  h. Perform RBC lysis as described in previous section.

### Flow cytometry staining

**Timing: 1–1.5 h**

This step demonstrates the surface staining of the spleen, thrombus, and bone marrow cells.

15. Surface staining.
  a. Transfer spleen, thrombus and bone marrow single cell suspensions into a round-bottom 96-well plate.
    **CRITICAL:** The number of cells should correspond to the antibody dilution. More cells could mean less antibody dilution.
  b. Centrifuge at 350 ×*g* for 5 min at 4°C.
  c. Discard supernatant and resuspend pellet in 100 μL Fc Blocking solution (1:50 dilution in FACS buffer).
  d. Incubate the plate in dark at 4°C for 20 min.
    ***Note:*** Prepare the antibody (Table S2) cocktail (1:200 dilution) during this incubation step. Protect from light and store on ice.
  e. Wash the plate with 100 μL FACS buffer and centrifuge at 350 ×*g* for 5 min at 4°C.
  f. Discard the supernatant and resuspend the pellet in 100 μL surface staining antibody cocktail.
  g. Incubate the plate in the dark at 4°C for at least 30 min.
  h. Wash with 100 μL FACS buffer and centrifuge at 350 ×*g* for 5 min at 4°C.
  i. Resuspend pellets in 200 μL FACS buffer and transfer to 5 mL FACS tubes for acquisition on BD LSRII.
    ***Note:*** Make sure to have compensation and FMO controls for the whole panel. Unstained and single-stained controls are important to avoid the overlap of fluorochromes. FMO controls help define gating and reduce false positives.

## Expected outcomes

This protocol enables reproducible induction of venous thrombus formation in C57BL/6 J mice using partial IVC stenosis, non-invasive assessment of thrombus formation by HFUS at 48 h post-ligation, and quantification of thrombus-infiltrating myeloid cells using flow cytometry. At day 2 after stenosis, a cylindrical and non-compressible thrombus is typically observed distal to the ligation site. HFUS reveal restricted venous blood flow with preserved abdominal aortic flow.

**Figure 4:**
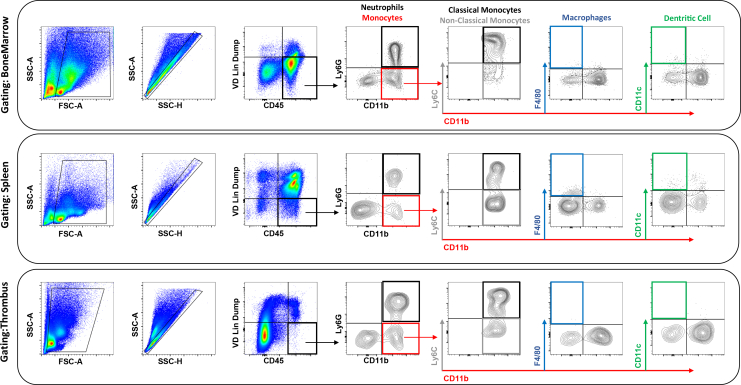
Frequency and gating strategy for Neutrophils, monocytes, macrophages and dendritic cell population in bone marrow, spleen, and thrombus

**Figure 5:**
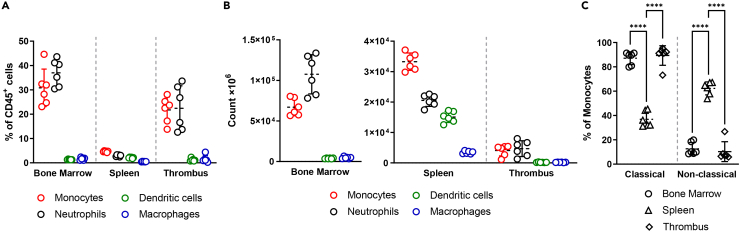
Flow cytometric profiling of myeloid cell populations (A) Myeloid cell populations plotted as frequency of CD45^+^ cells. (B) Cell populations were shown in absolute cell counts. (C) Monocyte subsets (classical and non-classical) were defined with Ly6C expression. Statistical significance was determined by two-way ANOVA followed by Tukey’s multiple comparisons test. Data are presented as mean ± SD. P value < 0.05 was considered statistically significant.

Single cell suspensions isolated from thrombi at day 2 typically yield 3–5 × 10^5^ viable cells per thrombus, depending on mouse weight, clot size, and surgical precision. Myeloid cell population frequencies are gated on (Figure 4) and reported as percentage of live CD45^+^ cells (Figure 5A) or absolute cell counts (Figure 5B). Monocytes and neutrophils represent the major myeloid populations present at this early timepoint, with classical Ly6C^+^ monocytes reliably detectable within the CD45^+^ leukocyte compartment (Figure 5C). However, the pan-dendritic and macrophage cell populations are barely recruited at the site of thrombosis. Expected viability after processing is typically >70%.

## Quantification and statistical analysis

Manual thrombus measurements (area, length, width) should be exported from Vevo software and analyzed in Prism or R. Biological replicates (independent mice) rather than technical replicates should be used as n for statistical analysis. Perform a minimum of 3 independent experiments to ensure reproducibility. For flow cytometry analysis, the data was obtained from independent biological replicates (n = 5–6). Statistical analysis was conducted using GraphPad Prism. Comparison of classical and non-classical monocytes across bone marrow, spleen and thrombus was performed. Statistical significance was determined by two-way ANOVA followed by Tukey’s multiple comparisons test. Data are presented as mean ± SD. P value < 0.05 was considered statistically significant.

## Limitations

Although this protocol provides a robust and reproducible method to induce venous thrombosis using partial IVC stenosis, several limitations should be considered. First, thrombus size and incidence remain sensitive to subtle variations in surgical technique and inter-animal anatomical differences. In particular, the spatial distribution of IVC side branches represents a major source of variability, as branches located in close proximity to the stenosis site can significantly alter local hemodynamics and impair thrombus formation. Second, thrombus composition at day 2 primarily reflects early inflammatory recruitment and may not fully capture later stages of thrombus organization or resolution. Third, anatomical variation in IVC side branches may differ by sex and strain, contributing to variability in thrombus size and cellular yield despite standardized procedures. Therefore, sample size should be determined empirically for each experimental setting, and anatomical features should be documented whenever possible. Finally, thrombus cell yields are inherently low and highly sensitive to processing time and mechanical handling. Excessive manipulation during isolation can affect immune cell viability and activation states. To enhance reproducibility and transparency, outcomes should be reported in a sex-stratified manner and, where feasible, include documentation of IVC branch anatomy.

## Troubleshooting

### Problem 1

No thrombus detection after 48 h. (Step 3).

### Potential solution

The thrombus might not form because of incomplete stenosis.

• Re-check ligature tightness after finishing the ligation.
• Use of consistent spacer gauge can help with consistent stenosis.
• Blood flow restriction can be verified by the dilation of the vein below the ligation site.
• Bleeding of IVC during surgery can perforate the vein wall preventing blood flow restriction and hypoxic conditions.
• Right side branch ligation leads to better thrombus formation.
• Mouse sex, age, and strain can affect thrombus formation. Increase sample size to confirm the validity of changes in thrombus size.

### Problem 2

Post-surgical mortality within the recovery period. (Step 6).

### Potential solution

• Detection of the gap between the vein and artery below the left renal vein is crucial. Failure to identify this gap can lead to ligation of vein and artery together leading to obstruction in the essential blood circulation.
• Prevent hypothermia using heating pad or infrared light whenever needed.
• Schedule analgesia treatment properly to avoid pain and stress.

### Problem 3

Postoperative infection/sepsis (Step 2, Step 5).

### Potential solution

• Do not damage the intestines. Perforation can leak gut bacteria causing infection in the abdominal cavity.
• Avoid non-sterile instruments, gloves, or environment.
• Close the wound properly to prevent bacterial entry.

### Problem 4

Low cell yield and viability of isolated cells from thrombus. (Step 10).

### Potential solution

• Thrombus can break easily. During extraction, use forceps to drive it out of the open end of vein. It helps keep thrombus intact.
• Perform the isolation fast and transfer the thrombus immediately into the ice-cold FACS buffer.
• Keep the thrombus extraction and cell isolation time to minimum.

### Problem 5

Overlapping fluorescence of different fluorochromes and difficulty in gating. (Step 15).

### Potential solution

• Run single stained controls before acquiring the samples.
• Optimize PMT voltage using these single stained controls.
• Prepare FMO controls to ensure proper gating of different cell populations or detect auto-fluorescence.

## Resource availability

### Lead contact

Further information and requests for resources and reagents should be directed to and will be fulfilled by the lead contact, Fatemeh Shahneh (fatemeh.zare-shahneh@unimedizin-mainz.de).

### Technical contact

Technical questions on executing this protocol should be directed to and will be answered by Aman Damara (damaraam@uni-mainz.de).

### Materials availability

This study did not generate new unique materials. Mouse strains used in this protocol are commercially available or can be requested via lead contact, where restrictions apply.

### Data and code availability

This protocol does not generate large-scale datasets or custom code. Raw thrombus measurements, HFUS imaging files, and flow cytometry acquisition files referenced in this manuscript will be made available upon reasonable request to the lead contact and technical contact.

## Acknowledgments

We acknowledge the CTH platforms and the Animal Facility of the University Medical Center Mainz for technical support. This work was supported by the 10.13039/501100001659German Research Foundation (10.13039/501100001659Deutsche Forschungsgemeinschaft, 10.13039/501100001659DFG) under grant ZA 1247/1-1 (project no. 507777753). The graphical abstract created using Biorender.com.

## Author contributions

A.D. performed most experiments, analyzed data, and wrote the manuscript. M.L.B. performed HFUS and contributed to data analysis. K.S. provided expertise and contributed to manuscript writing. F.S. conceptualized and coordinated the study and wrote the manuscript with input from all authors.

## Declaration of interests

The authors declare no competing interests.
