# Supplementary material for: Protocol for inducing deep vein thrombosis in C57BL/6J mice using the inferior vena cava stenosis model
Source: STAR Protoc. 2026 Jul 2;7(3):104665. doi: 10.1016/j.xpro.2026.104665 (PMC13352384; doi:10.1016/j.xpro.2026.104665)
Supplement: Document S1. Tables S1 and S2 [file mmc1.pdf]

Supplementary File

Table 1: Standardized post-surgery behavioral scoring sheet for monitoring mice, related to step 6.

Category	Condition
**Body Weight (relative to baseline)**	Unaffected or increased
	Reduction of 10–20%
	Reduction > 20%
**General Condition**	Coat smooth/shiny/lying flat; eyes shiny; body openings clean
	Coat dull; eyes cloudy; hunched posture; increased respiratory rate
	Coat rough; eyes sunken/cloudy; sticky or moist body openings; labored breathing
	Coat bristled; animal cold; eyes closed; seizures; paralysis; respiratory noises; bluish mucous membranes; bleeding from body opening
**Spontaneous Behavior**	Alert, curious, rearing, quick movements
	Unusual behavior; limited movement; hyperactivity; reduced exploratory behavior
	Isolation; apathy; coordination disorders; pronounced stereotypies or hyperkinesia
	Auto mutilation, necrosis, rectal prolapse
**Experiment-Specific Criteria (During Surgery)**	Cannot establish seal of jugular vein catheter
	Uncontrollable bleeding
	Injury to abdominal organs
**Experiment-Specific Criteria (After Surgery)**	Slightly reddened suture or injection sites
	Clearly reddened suture or injection sites
	Surgical wound slightly reddened, dry
	Wound edges clearly swollen; serous discharge
	Significant bleeding immediately after surgery
	Chewing/opening of surgical suture by the animal
	Opening of surgical suture (even after repair under anesthesia)
	Prolapse of intestinal segments through surgical wound
	Cooling of hind limbs; hind limb paralysis (due to circulatory disturbance)

Table 2: Antibody panel for flow cytometry detection of myeloid populations, related to step 15.

Cell Marker	Fluorochrome
CD11b	APC
Lineage (CD3, CD45R, NK1.1), Fixable viability dye	BV510
Ly6G	FITC
CD45	BV711
Ly6C	PE-Cy7
F4/80	BV421
CD11c	PerCP-Cy5.5
